# Remarks upon Ciliary Blepharitis

**Published:** 1857-12

**Authors:** W. F. Westmoreland

**Affiliations:** Prof. of Surgery in the Atlanta Medical College


ATLANTA

Medical and Surgical Journal.

VOL. III.] DECEMBER, 1857. [NO. IV.

### ORIGINAL COMMUNICATIONS.

### ARTICLE I.

*Remarks upon Ciliary Blepharitis* By W. F. WESTMORELAND, M. D., Prof. of Surgery in the Atlanta Medical College.

Within the past few years, it has been our fortune to witness quite a number of cases of this rebellious affection, in its several stages, and of observing its unsightly, and in some instances, irreparable results. As there is, perhaps, no disease which at first appears so simple in character, that gives the physician so much trouble by its frequent relapses, and unpleasant complications, we have thought it would not be amiss to direct the attention of the profession to it by giving the result of our observation and investigation as to its character and treatment.

By Ciliary Blepharitis we mean that disease of the eye in which the free borders of the lids are, at first, alone inflamed —the sebaceous glands which open in the follicles of the eye lashes, and the glands of Meibomius being first attacked. Some writers make several divisions and sub-divi ions of this affection, which rather tend to confuse than otherwise—their divisions having for basis the set of glands first involved in the inflammation, the peculiarity of symptoms as presented in different constitutions, or in the different stages of the disease.

We shall not stop here to discuss the propriety of these divisions, believing, as we do, that if one set of glands are attacked, the other, if the affection progresses, is soon involved, the disease running the same course, and presenting the same symptoms and complications in both. Much confusion, too, has resulted from the multiplicity of names under which it has been described. Beer, who has written a most excellent treatise upon this lesion, remarks that he knows of no disease which presents more confusion in regard to name than the one under consideration. Thus it has been described as *Ophthalmia Tarsi, Glandulous Ophthalmia, Dry Blepharitis, Scrofulous Ophthalmia, Blepharite Tarsi, Tarsal Inflammation, &c., &c.:* each author adopting a name in accordance with his peculiar views as to its character, or which best suited his fancy. To prevent confusion in noting the symptoms and complications consequent upon the extension of the disease, we have thought it best to make three stages, or degrees of intensity.

*First Stage.*—In the most simple form of Ciliary Blepharitis, the sufferings of the patient are very slight; and if the lids be well cleansed, it is with difficulty that the physician can detect any lesion whatever; the only symptoms being small scales attached to the base of the half dozen or more eyelashes, with a stiffness and smarting sensation when the patient has been exposed for any length of time to an artificial light, bleak wind, or such other cause as will tend to irritate the part. These thin scales are the result of a super-secretion of the glands before alluded to, the watery contents of this puriform collection evaporating as soon as it comes in contact with the free surface. One of the peculiarities of this squamiform substance is, that in removing it with a probe we find that it passes over the whole length of the eyelashes. It is not unusual to find them, if great care be not taken to keep them removed, entirely detached from the lid and resting midway between the base and the ends of the eyelashes by which they are pierced. Corresponding with the follicles diseased we usually find a slight tumefaction of the lid, and if the disease has existed some length of time, or if there be much irritation of the parts from accidental causes above suggested, that portion of the lids tumefied becomes more or less red, and in some cases vessels can be traced running from the base towards the free borders of the lids.

The above are the symptoms usually observed in the most simple form of the disease. As the disease progresses all these symptoms are increased. From a mere point upon one of the lids the inflammation extends to the whole free border of the lids, all the glands and hair follicles being involved in the inflammation. The patient now suffers greatly from the least exposure to causes that will tend to irritate the part; the lids are tumefied and red, and it is not unusual to find small ulcers along the free borders. The eyelashes from the very commencement of the disease, fall out, but, at first, are reproduced; the little ulcers above alluded to, usually occupying two or three hair follicles. The ulcers are seldom perceptible until the secretions are removed, and the parts thoroughly cleansed.

We have thus been particular in describing the first stage or degree of intensity, as it is in this stage that the disease most readily yields to treatment.

*Second Stage.*—It is in the second stage that the physician is most frequently consulted, and then not so much for the original disease, as for accidents and complications consequent upon its extension. In this stage, it is not confined to the free border of the eye-lids, but extends to the conjunctiva, first to that portion of the membrane forming the internal layer of the lids, and next to that reflected upon the ball; not unfrequently, as will be seen hereafter, the cornea becomes involved in the inflammation. As soon as the mucous membrane becomes inflamed, the secretions are changed in character, and are much more profuse—presenting the same appearance, so far as this feature is concerned, as is usually observed in Catarrhal Ophthalmia. The ulcers, before alluded to, on the borders of the lids, are more numerous, larger and deeper; the tumefaction of the lids is increased; and if the secretion be profuse it is not unusual to see slight abrasions or ulcers upon the lids. If the mucous membrane lining the lids be slightly tumefied at the same time that these ulcers exist, there will be a slight deviation of the lids—a disposition to ectropion, which, if the disease continue uninterrupted, will result in a complete eversion of one or both lids. Another accident, common to this stage, which is annoying, both to physician and patient, is a deviation of the puncta lachrymalia, the tears which are copious from the irritation about the eye, mixed with the other secretions, pass over the cheek, and sometimes excoriate it to such an extent that it is one of the greatest sources of suffering to the patient. This deviation of the puncta often takes place before there is any perceptible deviation of the lids. The inflammation, in some cases, extends to the lachrymal canals, even in the commencement of this stage, and often, notwithstanding the arrest of the original affection, continues until a lachrymal, tumour, or fistula is the result. Scarpa contends that this is one of the most frequent sources of lachrymal fistula. Whether this complication is the result of an extension of the inflammation to the canals and sac by continuity of surface; or, whether, as Scarpa supposed, it is the result of the passage of the tears mixed with the purulent secretions through this their natural channel, we will not stop here to discuss. Suffice it to say, that it is a frequent and very troublesome accident. Frequent crops of styes, which we neglected to mention as a symptom of the most simple form of the disease, are also common to this stage. It is in patients with a Scrofulous diathesis that this little accident is most frequently observed.

As above suggested, it is in this stage that the physician is most frequently consulted, not so much, however, for the original disease as the accidents, which are the principal source of their suffering. The physician, too, often commits the grave error of prescribing for the complications which most annoy the patient, without any regard whatever, to the original affection; the consequences being, that the immediate sufferings of the patient are relieved, to return again in a few weeks, or months, with more intensity.

In the third stage, all the symptoms enumerated are greatly aggravated; the eyelashes are all destroyed, or if there reremains a few, they are irregular and unhealthy. The ulcers upon the borders of the lids are much larger and more numerous; in some cases, the whole of the free borders of the lids are ulcerated, and so extensive at some points, that the tarsal cartilages are, to a great extent, destroyed. In some cases, there are extensive and repeated abcesses of the lids, if upon the outside, ectropion is the invariable result; if, however, the abcess bursts upon the mucous surface—the inside of the lid —Trichiasis, with all its unpleasant consequences, is the result. In more than one case, we have witnessed a destruction of the cornea, with a total loss of vision, from an inflammation of the conjunctiva and cornea, induced by this unpleasant deformity. In the absence of any abrasion or ulceration, there are cases in which we see, both in the second and third degrees of intensity, a contraction of the skin under the inferior lid, inducing a kind of ectropion, which, without a careful examination, would appear to be the result of an inflammation and thickening of the mucous membrane lining the lids. From this mistake patients are sometimes forced to submit to the pain of an excision or cauterization of the thickened mucous membrane, without any favorable result, when the deformity is readily corrected, by the application of unguents, or such other remedies as will protect the skin from the irritating effect of the tears and purulent secretions which pass over it, and which, alone, induces this deviation of the lid.

There are other accidents, of which we would like to speak, but if we were to attempt to give, in detail, all the unpleasant complications that may result from this, at first very simple lesion, we would extend this article far beyond the limits first intended.

The course of the disease, particularly in the first stage, is decidedly chronic, the most simple form existing, even without treatment, for months, and even years. The second and third degrees, however, are more acute in character, accidents or complications arising in both, which, if not met with appropriate remedies, will result in a destruction of the eye, or in some unpleasant deformity.

*Treatment.*—As the first great indication in the treatment of this, as in all other diseases, is to remove the cause, it is important, before speaking of the remedies most applicable, to notice, to some extent, at least, some of the sources of the disease. The Scrofulous diathesis is one of the most prominent predisposing causes; so frequent is it that some writers contend that it is alone in this class of subjects that we see the disease—consequently the name *Scrofulous Ophthalmia.* We are, however, convinced from our own experience, as well as the experience of those high in authority, that there are quite a number of cases in which we cannot detect the least taint of this unfortunate diathesis. The want of a sufficiency of digestible food, impure atmosphere, intemperance, &c., are said to be predisposing causes of this disease. The exciting causes are numerous, and are usually such as tend to irritate and congest the eye. Several of the professions are exciting causes, thus the stone-dresser, baker, plasterer, practical chemist, &c., are frequent subjects of this affection. Exposure to artifical light, for a length of time, as reading at night, or, as in some of the professions, as the jeweler, is a frequent exciting cause of the disease.

The first indication, then, in whatever stage we may be called to treat the affection, is, to remove the exciting cause. This is not, however, so readily accomplished as we would at first imagine, particularly in the first stage, as in this the most simple form, it is difficult to induce the patient to give up for the time, his occupation for what he considers such an unimportant affection. If our patient is of lymphatic temperament and disposed to Scrofula, such remedies as are applicable in this peculiar affection must be used; if there be other predisposing causes, as above suggested, such a hygeinic course as will tend to change this constitutional defect must be adopted.

If we are called to treat the disease in its first stage, our local treatment will, at first, consist of such applications as will remove the scales found at the base of the eyelashes, and, at the same time, allay the irritability of the part inflamed. We have no application that acts so happily as a small poultice made of well cooked rice, moistened with fresh milk. If this cannot be readily procured, we may substitute a poultice made of ground flax seed. It is seldom that we can induce the patient to apply the poultice during the day; if they are applied at night they should be renewed once or twice, during the night. The poultice softens the secretion so that the scales may be readily removed with a hair-pin, probe, or in some cases with tepid water and a fine linen cloth. It is of the very greatest importance that the scales be removed, as they are a source of great irritation, and at the same time, effectually prevent any remedial agent coming in contact with the diseased surface. After we have, in this way, thoroughly cleansed the part, we will be able to determine the extent of the lesion; if there be small ulcers, which we sometimes find even in the commencement of the disease, they should be touched lightly with a stick of the nitrate of silver; or, if there be no ulcers, the border of the lid may be touched by means of a small camel's hair brush, with a solution of nitrate of silver—twenty grains to the ounce. Numerous ointments have been proposed here, as the red precipitate of various strengths, ointments made of the nitrate of silver, white oxide of lead, &c., &c. The application of the stick of the sulphate of copper has, also, been suggested and practised with some success. But whatever agent we may select, it is of the very greatest importance that the secretions be removed, and the diseased surface thoroughly cleansed, before their application. The poultices should be renewed from time to time, as circumstances may demand. The patient should be impressed with the importance of removing the secretions as soon as they make their appearance. In impressing this feature of the treatment upon his class, I often heard M. Desmarres remark, during the past winter, that he never had much difficulty in arresting this affection in young ladies who were disposed to be coquettish, as they, for appearance sake, if nothing more, would remove the secretions as fast as they would form. He contends that he has often arrested the disease by removing the cause, the application of the poultice, and great care in removing the secretions as soon as formed. If we use the stick or the solution of the nitrate of silver, it is best to apply a little simple cerate or olive oil to the borders of the lid, thus keeping them soft—preventing that hardening which so often results in cracks and excoriations. Astringent eye-washes are worse than useless, in this form of the disease. In the second stage, the treatment, so far as regards the original affection, differs but little, if any, from that just described. If the conjunctiva is inflamed, slight astringent washes, with soothing applications, in addition to that suggested for the more simple form, is all that will be necessary.

If there be excoriations and ulcers upon the lids, from the contact of the secretions, the part must be protected, which may be effectually done by covering the ulcers or excoriations with simple cerate, or some soothing ointment. This precaution will often prevent a deviation of the puncta lachrymalia; and, in other cases, will relieve that disposition to ectropion which we described as one of the accidents of the second and third degrees. Cleanliness, if you will allow the expression, is more important in this stage than in the first.

In the third, the ulcers, as before said, are much larger, deeper, and more numerous than in the other two degrees; the application of the stick of the nitrate of silver, after the ulcers have been well cleansed, is the most effectual way to arrest their progress. To reach the bottom of the ulcers, which is important, it is necessary to trim the stick to a point. The cauterization of their edges, would do but little towards removing the disease. Poultices in this stage are, at first, of the very greatest importance, as there is always much irritation about the eye. Trichiasis, ectropion, lachrymal fistula, &c., usually require an operation for their relief.

